# Impact of obesity on reduction quality and operative outcomes of proximal femoral nail for intertrochanteric femoral fractures in the lateral decubitus position

**DOI:** 10.1186/s13018-026-07301-5

**Published:** 2026-10-07

**Authors:** Emre Teksan, Halil Karaca

**Affiliations:** 1 Department of Orthopaedics and Traumatology, Giresun University, Giresun, Turkey; 2 Department of Orthopaedics and Traumatology, Arhavi State Hospital, Artvin, Turkey

**Keywords:** Intertrochanteric femoral fracture, Femoral nail, Obesity, Complication

## Abstract

**Background:**

The aim of this study is to compare the clinical and radiological outcomes of proximal femoral nailing performed in the lateral decubitus position between obese and non-obese patients.

**Methods:**

This retrospective cohort study, approved by the institutional ethics committee, included patients with acute AO/OTA classification types 31A1, 31A2, and 31A3 intertrochanteric femoral fractures treated with closed reduction and osteosynthesis using a short proximal femoral nail between 2022 and 2024. Obesity was defined as a body mass index of ≥ 30 kg/m^2^. Patients were divided into obese and non-obese groups, and clinical, radiological, and complication outcomes were compared between the two groups.

**Results:**

A total of 533 patients were included, comprising 95 obese and 438 non-obese individuals. Obese patients were significantly younger and had higher Charlson Comorbidity Index scores (*p* < 0.001). Operative time and intensive care unit admission rates were significantly higher in the obese group (*p* < 0.001), and anesthesia type distribution differed between groups (*p* < 0.001). Length of hospital stay and Harris Hip Scores were comparable (*p* > 0.05). Radiologically, the distribution of tip–apex distance according to the 25-mm threshold did not differ between groups (*p* = 0.244). However, optimal screw placement according to the Cleveland index was significantly less frequent in obese patients (*p* < 0.001), and malposition in the lateral view was more common (*p* < 0.001). Varus alignment and differences in reduction quality were also significantly associated with obesity status (*p* < 0.001). Multivariable logistic regression analysis demonstrated that obesity remained an independent predictor of postoperative varus malalignment (OR 2.80, 95% CI 1.58–4.96; *p* < 0.001).

**Conclusion:**

Obesity negatively affects reduction quality and operative parameters during proximal femoral nailing for intertrochanteric femoral fractures performed in the lateral decubitus position, although acceptable reductions can generally still be achieved. Furthermore, obesity remained an independent predictor of postoperative varus malalignment after adjustment for potential confounding factors. Surgeons should be aware of these technical challenges when treating obese patients and consider them during preoperative planning and intraoperative reduction maneuvers.

## Introduction

Intertrochanteric femoral fractures (IFFs) account for approximately half of all hip fractures and represent a major public health concern, particularly in the elderly population [1, 2]. These fractures are associated with substantial morbidity, mortality, functional decline, and reduced quality of life [3]. Therefore, achieving stable fixation and appropriate fracture reduction is essential to enable early mobilization and minimize postoperative complications.

Proximal femoral nail (PFN) has become one of the most widely preferred fixation methods for IFFs because of its biomechanical advantages and lower complication rates compared with other implant options [4]. However, obtaining adequate reduction and optimal implant positioning may be technically challenging in certain patient populations.

Obesity is a growing global health problem associated with increased perioperative difficulty, higher complication rates, and impaired surgical visualization due to excessive soft tissue thickness [5]. Despite the increasing prevalence of both obesity and hip fractures, limited evidence exists regarding the impact of obesity on radiological and clinical outcomes following PFN for IFFs.

Therefore, the aim of this study was to compare the clinical and radiological outcomes of PFN performed in the lateral decubitus position between obese and non-obese patients with intertrochanteric femoral fractures.

## Methods

This study was conducted using a retrospective cohort design and received approval from the institutional ethics committee. All study procedures were performed in accordance with the ethical standards outlined in the Declaration of Helsinki, and informed consent was obtained from all participants.

Patients were included if they had acute AO/OTA (AO Foundation/Orthopaedic Trauma Association) classification types 31A1, 31A2, and 31A3 intertrochanteric femoral fractures resulting from low-energy trauma and underwent closed reduction and osteosynthesis with a short PFN between 2022 and 2024. Patients with AO/OTA 31A1, 31A2, and 31A3 fracture patterns were included if they were managed using the same closed reduction and short PFN fixation protocol in the lateral decubitus position. Fracture type was recorded for all patients and included in the multivariable regression model to account for potential differences in fracture complexity. The exclusion criteria were as follows: pathologic fractures, open fractures, open reduction performed during surgery, the use of additional implants, polytrauma, and incomplete demographic data or radiographs.

**Fig. 1:**
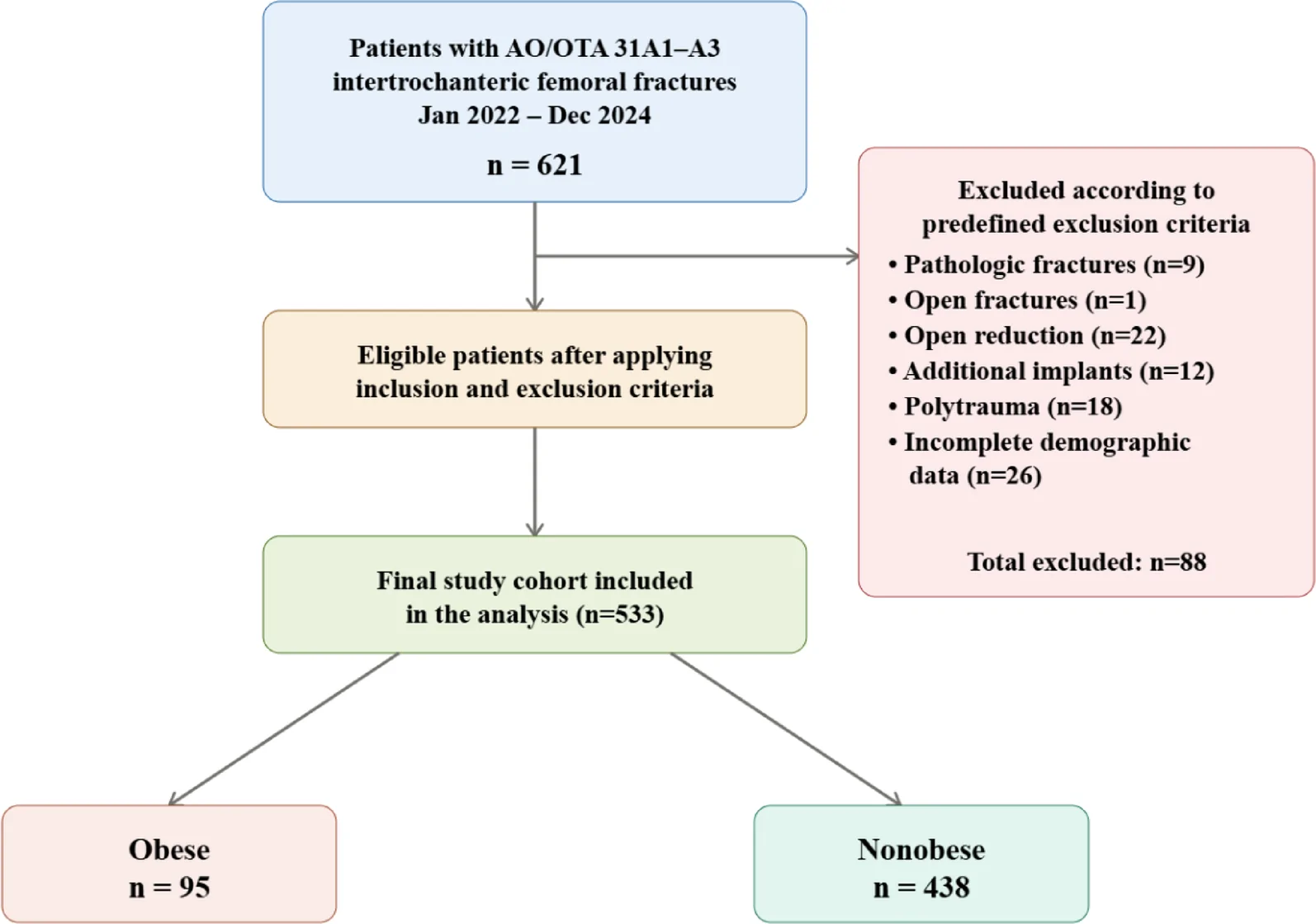
Flow diagram illustrating patient selection and application of inclusion and exclusion criteria

Patients who required open reduction were excluded because the primary aim of the study was to evaluate the technical challenges and radiographic outcomes of closed reduction and cephalomedullary nailing under standardized operative conditions. Inclusion of open reduction cases could have introduced additional variability related to reduction techniques and intraoperative decision-making. The patient selection process is summarized in Fig. 1.

Body weight classification was performed using body mass index (BMI). Accordingly, a BMI of ≥ 30 kg/m^2^ was defined as obesity. Based on this criterion, patients included in the study were categorized into two groups: obese (BMI ≥ 30 kg/m^2^) and non-obese (BMI < 30 kg/m^2^), and were analyzed accordingly.

Assessment data were obtained from the hospital database, hospital electronic imaging system, and patient-reported outcome measures.

### Follow-up and functional assessment

Patients were routinely followed at 2 weeks, 6 weeks, 3 months, and 6 months after surgery. The mean follow-up duration was 13.2 ± 5.8 months (range, 6–24 months). Prefracture functional status was assessed using the Parker Mobility Score, based on information obtained from the patient, relatives, or medical records at admission. Harris Hip Scores were assessed at the final outpatient follow-up. All patients included in the functional outcome analysis had a minimum follow-up of 6 months.

*Parameters obtained from the hospital database*; the analyzed parameters included patient age, sex, laterality, body mass index, intensive care unit (ICU) admission requirement, length of hospital stay, operative time, type of anesthesia, preoperative and postoperative osteoporosis treatment history, and Charlson Comorbidity Index (CCI).

*Parameters obtained from the electronic imaging system*; Radiographs were reviewed by two orthopedic surgeons using the institution’s picture archiving and communication system. When discrepancies occurred between the two observers regarding radiographic classifications, the images were re-evaluated collaboratively until consensus was achieved. All trauma-related images from the initial presentation were included in the evaluation; anteroposterior pelvic and cross-table lateral radiographs were assessed.

The analyzed parameters included AO/OTA classification, tip–apex distance (TAD), caput-collum-diaphyseal angle (CCDA), screw position, postoperative implant-related complications, including screw penetration to the hip joint (cut-out), implant fracture, and Modified Baumgaertner’s reduction criteria. Postoperative implant-related complications included screw penetration to the hip joint (cut-out), implant fracture, Z-effect, and V-effect–related varus malposition. V-effect–related varus malposition was defined as postoperative varus malalignment associated with eccentric nail position and inferior displacement of the cephalic fixation construct, resulting in a characteristic V-shaped radiographic appearance. In the dual-screw PFN system used in this study, Z-effect was defined as differential migration of the two proximal cephalic fixation screws in opposite directions within the femoral head-neck segment, resulting in loss of proximal fixation.

Postoperative alignment was grouped into varus (CCDA < 125°), neutral (125°–135°) and valgus (CCDA > 135°). We considered varus postoperative alignment as improper and neutral or valgus postoperative alignment as proper [6].

Adequacy of reduction was categorized into good, acceptable or poor reduction as described by Baumgaertner et al. [7]. Two criteria were evaluated. The first criterion used was a CCDA neutral (125–135°) and a lateral angulation of < 20°. The second criterion used was the presence of < 4 mm of displacement of any fragments in the AP and lateral views. Reduction was considered as good if both criteria were met, acceptable if only one criterion was met and poor if neither of the criteria was met.

Screw position within the femoral head was evaluated on postoperative radiographs using the Cleveland method, which divides the head into three zones (superior, central, inferior) on the anteroposterior view and three zones (anterior, central, posterior) on the lateral view, resulting in nine possible locations. The central-central and inferior-central placement of the implant within the femoral head was considered the optimal position [8]. In cases where the implant position was located at the border between two Cleveland zones, classification was assigned according to the zone containing the greater proportion of the implant. When uncertainty existed, the final classification was determined by consensus between the two reviewers.

**Fig. 2:**
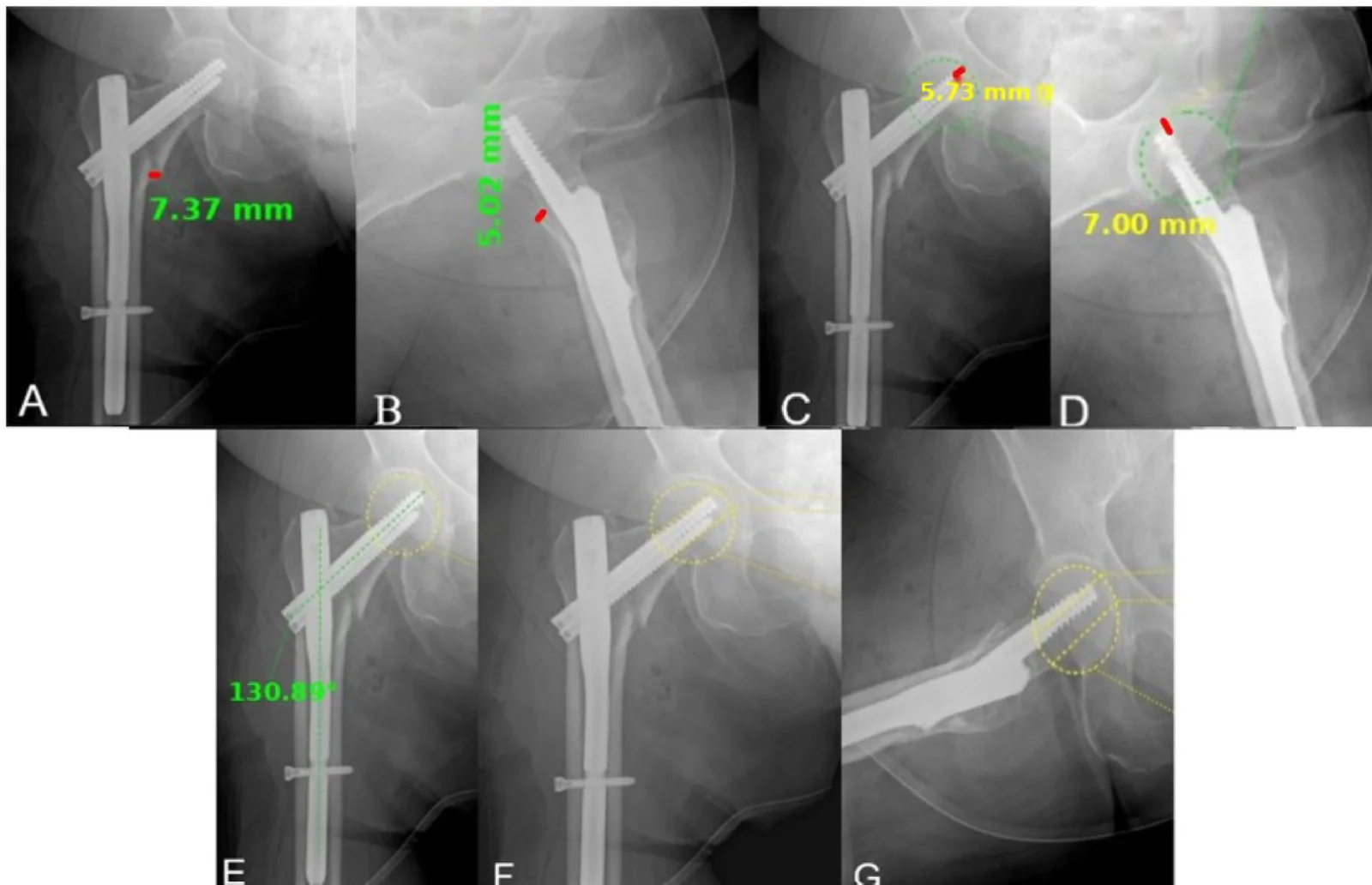
Radiographic assessment of fracture reduction quality and implant position following proximal femoral nail. **A**, **B** Measurement of calcar fragment displacement on anteroposterior and lateral radiographs; **C**, **D** tip-apex distance measurements obtained on anteroposterior and lateral views; **E** measurement of the caput-collum-diaphyseal angle; and **F**, **G** evaluation of implant position within the femoral head on anteroposterior and lateral radiographs

TAD was measured on postoperative anteroposterior and lateral radiographs, calculated as the sum of the magnification-corrected distances from the implant tip to the femoral head apex, and categorized as < 25 mm or ≥ 25 mm (Fig. 2).

Clinical outcomes included Harris Hip Score assessment and the evaluation of fracture union status. Nonunion was defined as the absence of radiographic progression of fracture healing and persistent fracture lines at a minimum of 6 months after surgery, accompanied by persistent pain during weight bearing.

*Preoperative preparation*: To prevent venous thromboembolism, low-molecular-weight heparin prophylaxis was initiated in all patients upon hospital admission. The prophylactic regimen was discontinued 12 h prior to surgery and resumed 6 h postoperatively. As part of the preoperative assessment, all patients underwent chest radiography, electrocardiography, complete blood count, electrolyte analysis, liver and renal function tests, blood typing, coagulation studies, and required serological examinations. If patients had hemoglobin level lower than 9 g/dl, patients were transfused preoperatively to at least this level. Relevant specialty consultations were obtained for existing comorbidities, and patients were evaluated by the anesthesia team according to the American Society of Anesthesiologists classification. Surgical intervention was performed only in patients deemed suitable for both surgery and anesthesia.

The decision for postoperative ICU admission was made by the anesthesia team based on the patient’s preoperative anesthetic evaluation and perioperative clinical condition. ICU admission was not routine and was reserved for selected patients who required closer postoperative monitoring. No predefined study-specific ICU admission criteria were applied.

### Surgical technique

All surgical procedures were performed by experienced orthopedic trauma surgeons from the same institution. Although multiple surgeons were involved, all procedures were carried out by members of the same orthopedic trauma team using a standardized surgical technique and postoperative rehabilitation protocol. All patients received 1 g of parenteral cefuroxime sodium as prophylaxis 60 min prior to surgical incision.

Surgical procedures were performed under either general or regional anesthesia. The choice of anesthesia was determined by the attending anesthesiologist based on the patient’s preoperative anesthetic evaluation, comorbidities, and overall clinical condition. Regional anesthesia was preferred whenever feasible, whereas general anesthesia was used when regional anesthesia was contraindicated, unsuccessful, or considered more appropriate by the anesthesia team. All operations were carried out with patients positioned in the lateral decubitus position.

Closed reduction was performed under fluoroscopic guidance using manual traction applied by an assistant. A sterile support was placed between the lower extremities when necessary to facilitate valgus alignment and improve fracture reduction. Reduction was achieved through a combination of traction, rotation, and alignment maneuvers. In cases with persistent medial cortical displacement, a bone hook was introduced through the surgical incision beneath the femoral neck to restore medial cortical apposition while maintaining a minimally invasive approach. No percutaneous K-wire-assisted or formal open reduction techniques were used.

Following satisfactory fracture reduction, short PFN fixation was performed through a minimally invasive trochanteric entry approach. The entry point was established at the tip or slightly lateral to the greater trochanter under fluoroscopic guidance. After guide wire placement and canal preparation, the nail was inserted with appropriate depth and rotational alignment. The proximal guide wire was positioned centrally on anteroposterior and lateral views, and a temporary antirotation guide wire was used during implant insertion in unstable fractures when necessary. The lag screw and antirotation screw were inserted after measurement over the guide wires, and screw positions were confirmed fluoroscopically. One distal locking screw and an end cap were used in all patients. No drains were used. Operative time was defined as the interval from skin incision to completion of wound closure.

### Implant characteristics

All patients were treated using the same short proximal femoral nail (PFN) system (Otto Medical, Konya, Türkiye). The implant is a cephalomedullary nail system incorporating a compression screw and antirotation screw construct, with distal static or dynamic locking capability for the treatment of intertrochanteric femoral fractures.

### Postoperative rehabilitation

All patients received a standardized postoperative management protocol. Prophylactic antibiotic therapy consisting of intravenous cefazolin sodium (1 g, four times daily) was administered within the first 24 h after surgery. Thromboprophylaxis with enoxaparin was prescribed for 30 days postoperatively, with dosing adjusted according to each patient’s body mass index. Mobilization was initiated on the first postoperative day with sitting, standing, transfer training, protected walker-assisted ambulation, and quadriceps strengthening exercises under the supervision of the rehabilitation team. A progressive partial weight-bearing program was generally initiated two weeks after surgery according to the patient’s clinical condition, pain tolerance, and postoperative stability. For patients managed in the intensive care unit, passive range-of-motion exercises were administered in bed by a physiotherapist. Ambulation and joint range-of-motion exercises were continued following transfer to the orthopedic ward.

### Statistical analysis

Statistical analyses were performed using IBM SPSS Statistics version 25.0 (IBM Corp., Armonk, NY, USA). Continuous variables were expressed as mean ± standard deviation, and categorical variables were expressed as frequencies and percentages. The normality of continuous variables was assessed using the Shapiro–Wilk test. Comparisons between obese and non-obese groups were performed using the independent-samples t-test or Mann–Whitney U test for continuous variables, and the chi-square test or Fisher’s exact test for categorical variables, as appropriate. There were no missing data for the analyzed variables in the final study cohort, and complete-case analysis was performed. Multivariable logistic regression analysis was performed to identify independent factors associated with postoperative varus malalignment. Variables included in the model were obesity status, age, sex, Charlson Comorbidity Index, ICU admission, AO/OTA fracture type, and anesthesia type. Odds ratios (ORs) with 95% confidence intervals (CIs) were calculated. A p value of <0.05 was considered statistically significant.

## Results

A total of 533 patients were included in the study, comprising 95 obese and 438 non-obese individuals. Body mass index (BMI) was significantly higher in the obese group (33.01 ± 3.40) compared with the non-obese group (25.79 ± 2.98) (*p* < 0.001). The obese patients were significantly younger than non-obese patients (78.47 ± 12.21 vs. 81.86 ± 12.56 years, *p* = 0.001). There was no significant difference between groups regarding sex distribution, fracture side, and AO/OTA fracture classification (*p* > 0.05).

Operative time was significantly longer in obese patients (109.42 ± 19.03 min) compared with non-obese patients (75.17 ± 21.74 min) (*p* < 0.001). Total length of hospital stay did not differ significantly between groups (6.96 ± 4.60 vs. 5.99 ± 7.20 days, *p* = 0.098).

The requirement for ICU admission was significantly higher in the obese group (*p* < 0.001). The type of anesthesia differed significantly between groups, with spinal/general anesthesia distribution of 72/23 in obese patients and 403/35 in non-obese patients (*p* < 0.001). There were no significant differences in preoperative osteoporosis treatment or postoperative initiation of osteoporosis treatment (*p* > 0.05).

Charlson Comorbidity Index (CCI) scores were significantly higher in obese patients (3.74 ± 1.45) compared with non-obese patients (3.00 ± 1.40) (*p* < 0.001). However, Harris Hip Score did not differ significantly between groups (78.16 ± 9.13 vs. 79.52 ± 8.28, *p* = 0.157). There was no significant difference in prefracture Parker Mobility Scores between obese and non-obese patients (*p* = 0.185) (Table 1).

**Table 1:** Comparison of demographic and clinical characteristics between obese and non-obese patients

	Obese (n = 95)	Non-obese (n = 438)	*p* value
Body mass index	33.01 ± 3.40	25.79 ± 2.98	**< 0.001**
Age	78.47 ± 12.21	81.86 ± 12.56	**0.001**
Gender			
Male	33 (34.7)	170 (38.8)	0.532
Female	62 (65.3)	268 (61.2)	
Fracture side			
Right	39 (41)	201 (45.8)	0.456
Left	56 (59)	237 (54.2)	
AO/OTA classification			
31 A1	24 (25.2)	142 (32.4)	0.389
31 A2	67 (70.5)	278 (63.5)	
31 A3	4 (4.3)	18 (4.1)	
Operative time	109.42 ± 19.03	75.17 ± 21.74	**< 0.001**
Total length of stay	6.96 ± 4.60	5.99 ± 7.20	0.098
ICU admission requirement	36 (37.9)	83 (18.9)	**< 0.001**
Anesthesia type			
Spinal	72 (75.8)	403 (92)	**< 0.001**
General	23 (24.2)	35 (8)	
Preoperative osteoporosis treatment (+)	17 (17.9)	92 (21)	0.589
Postoperative Osteoporosis Treatment Initiated	9 (9.5)	31 (7)	0.556
Charlson comorbidity index	3.74 ± 1.45	3.00 ± 1.40	**< 0.001**
Harris Hip Score	78.16 ± 9.13	79.52 ± 8.28	0.157
Parker Mobility Score	6.3 ± 3.0	5.9 ± 3.0	0.185

Data are presented as mean ± SD or n (%). The bold P values indicate significance. ICU; Intensive Care Unit, AO/OTA; AO Foundation/Orthopaedic Trauma Association, Significant at *P* < 0.05

There was no significant difference between groups regarding tip–apex distance (TAD) distribution (*p* = 0.244). However, significant differences were observed in Cleveland index classification. Optimal screw placement (cc/ci) was less frequent in obese patients (66.3%) compared with non-obese patients (87.2%), whereas non-optimal positioning was more common in the obese group (*p* < 0.001). In the anteroposterior view, screw position (central-inferior vs. superior) did not differ significantly between groups (*p* = 0.112). In contrast, screw position in the frog-leg lateral view showed a significant difference (*p* < 0.001), with obese patients demonstrating a higher proportion of anterior and posterior malposition. CCDA distribution differed significantly between groups (p < 0.001), with varus alignment more frequently observed in obese patients. Baumgaertner reduction quality was also significantly associated with obesity status (*p* < 0.001). Acceptable reductions were markedly more common among obese patients (Table 2).

**Table 2:** Radiographic and reduction-related parameters

	Obese (n = 95)	Non-obese (n = 438)	*P* value
TAD			
< 25 mm	86 (90.5)	411 (93.8)	0.244
≥ 25 mm	9 (9.5)	27 (6.2)	
Cleveland			
Optimal (cc/ci)	63 (66.3)	382 (87.2)	**< 0.001**
Non optimal	32 (33.7)	56 (12.8)	
Screw position (AP view)			
Central-inferior	89 (93.7)	427 (97.4)	0.112
Superior	6 (6.3)	11 (2.6)	
Screw position (Frog-leg view)			
Central	59 (62.1)	353 (80.5)	**< 0.001**
Anterior	13 (13.7)	51 (11.6)	
Posterior	23 (24.2)	34 (7.9)	
CCDA			
Varus	26 (27.4)	47 (10.7)	**< 0.001**
Neutral	66 (69.5)	384 (87.7)	
Valgus	3 (3.2)	7 (1.6)	
Baumgaertner reduction quality			
Good	42 (44.2)	353 (80.5)	**< 0.001**
Acceptable	48 (50.5)	55 (12.5)	
Poor	5 (5.3)	30 (7)	

Data are presented as n (%). The bold P values indicate significance. CCDA; caput-collum-diaphyseal angle, AP; anteroposterior, cc; central central, ci; central inferior Significant at *P* < 0.05

Superficial surgical site infection was significantly more frequent in obese patients than in non-obese patients (8/95, 8.4% vs. 11/438, 2.5%; *p* = 0.011). The incidence of nonunion did not differ significantly between groups (3.2% vs. 0.9%; *p* = 0.111). Screw penetration to the hip joint (cut-out) occurred significantly more often in obese patients than in non-obese patients (5/95, 5.3% vs. 3/438, 0.7%; *p* = 0.006). There was no significant difference in the incidence of Z-effect between obese and non-obese groups (3.2% vs. 1.6%; *p* = 0.370). Similarly, broken nail rates were comparable between groups (2/95, 2.1% vs. 2/438, 0.5%; *p* = 0.129). V-effect–related varus malposition was significantly more common among obese patients compared with non-obese patients (21/95, 22.1% vs. 32/438, 7.3%; *p* < 0.001) (Table 3).

**Table 3:** Postoperative complications

Complication	Obese	Non-obese	*p* value
Superficial surgical site infections	8 (8.4)	11 (2.5)	**0.011**
Nonunion	3 (3.2)	4 (0.9)	0.111
Screw penetration to the hip joint (cut-out)	5 (5.3)	3 (0.7)	0.006
Z-effect	3 (3.2)	7 (1.6)	0.370
Broken nail	2 (2.1)	2 (0.5)	0.129
V-effect–related varus malposition	21 (22.1)	32 (7.3)	**< 0.001**

Data are presented as n (%). The bold *P* values indicate significance. Significant at *P* < 0.05

Multivariable logistic regression analysis was performed to identify independent factors associated with postoperative varus malalignment. Obesity was independently associated with an increased risk of postoperative varus malalignment (OR 2.80, 95% CI 1.58–4.96; *p* < 0.001). General anesthesia was also independently associated with postoperative varus malalignment (OR 2.23, 95% CI 1.30–3.82; *p* = 0.004). In contrast, age, sex, Charlson Comorbidity Index, ICU admission, and AO/OTA fracture classification were not independently associated with postoperative varus malalignment (Table 4).

**Table 4:** Multivariable logistic regression analysis of ındependent factors associated with postoperative varus malalignment

Variable	OR	95% CI	*p* value
Obesity	2.80	1.58–4.96	**<** **0.001**
Age	1.00	0.97–1.02	0.656
Male sex	0.74	0.40–1.37	0.343
Charlson Comorbidity Index	0.85	0.71–1.03	0.096
ICU admission	0.94	0.52–1.70	0.830
General anesthesia	2.23	1.30–3.82	**0.004**
AO/OTA 31A2 vs 31A1	0.90	0.51–1.59	0.725
AO/OTA 31A3 vs 31A1	0.97	0.47–2.00	0.929

Data are presented as odds ratios (ORs) with 95% confidence intervals (CIs). Bold p values indicate statistical significance (*p* < 0.05)

OR, odds ratio; CI, confidence interval; ICU, intensive care unit; AO/OTA, AO Foundation/Orthopaedic Trauma Association

## Discussion

One of the main findings of this study is that achieving and maintaining adequate reduction during PFN performed in the lateral decubitus position is more technically demanding in obese patients. The specific contribution of the present study is that it evaluates the effect of obesity on reduction quality, implant positioning, and mechanical complications in a large cohort of patients treated with proximal femoral nailing in the lateral decubitus position, where manual traction rather than fracture-table traction is used. Increased soft tissue thickness in the peritrochanteric region may reduce the effectiveness of manual traction, impair fluoroscopic visualization, and complicate intraoperative alignment control [9–12].

In addition, obesity has been reported to negatively affect entry point control, implant positioning, and mechanical axis alignment during intramedullary nailing [13, 14]. In obese patients, despite achieving optimal screw placement on the anteroposterior view, obtaining optimal positioning on the frog-leg lateral view appears to be more difficult. Consistent with this finding, higher rates of varus malposition and suboptimal implant positioning were observed in obese patients. Possible explanations include inadequate intraoperative fluoroscopic image quality and difficulties in achieving sufficient anteroposterior reduction because of increased soft tissue around the hip. Furthermore, the use of manual traction may contribute to insufficient reduction control in these patients, potentially leading to varus malposition and suboptimal implant positioning.

The V-effect, first described by Hu et al., is an iatrogenic complication characterized by varus malalignment associated with eccentric nail insertion and inferior displacement of the cephalic fixation construct, resulting in a characteristic V-shaped radiographic appearance during PFN fixation of intertrochanteric femur fractures [15]. Previous studies have reported V-effect rates ranging from approximately 9.4% to 36.7%, potentially related to implant design and fracture characteristics [16, 17]. In our study, V-effect–related varus malposition was observed significantly more frequently in obese patients. Increased soft tissue resistance, difficulty identifying anatomical landmarks, and challenges in maintaining reduction may contribute to the development of this complication in obese individuals. In addition, Z-effect is a mechanical complication characterized by differential migration of the proximal fixation elements, which may result in loss of fixation and mechanical failure. Although Z-effect was observed in our cohort, its incidence did not differ significantly between obese and non-obese patients. Furthermore, multivariable logistic regression analysis confirmed that obesity remained an independent predictor of postoperative varus malalignment after adjustment for potential confounding factors. This finding further supports the association between obesity and reduction-related technical difficulties observed in the present study.

These findings emphasize the importance of meticulous reduction and careful intraoperative technique, particularly in high-risk patients. In obese patients, optimization of fluoroscopic imaging, adequate assistant-applied traction, careful control of limb rotation, and the use of sterile limb support may help improve reduction quality and implant positioning. In selected cases with persistent medial cortical displacement, limited use of adjunctive reduction tools, such as a bone hook introduced through the surgical incision, may facilitate restoration of medial cortical apposition while preserving a minimally invasive approach.

The literature reports that operative time is longer in obese patients compared to patients of normal weight [18]. Similarly, in our study, operative time was longer in obese patients. Possible explanations include difficulties at the femoral entry point, insufficient traction associated with manual traction techniques, loss of reduction after the frog-leg maneuver, and repeated reduction attempts. This situation lengthens the time after incision and potentially increases the risk of intraoperative infection. These findings emphasize the technical challenges of achieving and maintaining adequate reduction in obese patients and highlight the importance of careful intraoperative reduction maneuvers and fluoroscopic assessment.

Proximal femoral fractures are strongly associated with underlying osteoporosis and carry a high risk of secondary fractures [19–22]. Consistent with the literature, our study also demonstrated low rates of both preoperative and postoperative osteoporosis treatment. These findings emphasize the importance of systematic osteoporosis evaluation and timely initiation of anti-osteoporotic therapy in patients with proximal femoral fractures. Despite the well-established association between fragility fractures and osteoporosis, treatment rates remain suboptimal worldwide. Li et al. [23] demonstrated that inadequate osteoporosis treatment following hip fracture is associated with an increased risk of subsequent fragility fractures and poorer long-term outcomes. Therefore, hip fracture hospitalization represents an important opportunity for secondary fracture prevention through appropriate osteoporosis assessment and timely initiation of anti-osteoporotic therapy [23].

Obesity is often accompanied by various additional systemic diseases [24]. Therefore, in patients hospitalized due to hip fractures, preoperative preparation and postoperative hospital stay may be prolonged. Furthermore, these patients also have a greater need for intensive care [25]. The findings of our study support these observations and highlight the difficulties of treating hip fractures in elderly obese patients with comorbidities. Therefore, it is extremely important to identify the patient group at risk of complications and take preventive measures to reduce the burden of hip fractures on the healthcare system. In addition, the distribution of anesthesia type differed significantly between the study groups. This finding may reflect the greater technical difficulty of regional anesthesia and the increased frequency of medical comorbidities in obese patients, which may influence anesthetic decision-making in clinical practice. However, the present study was not designed to evaluate the independent effect of anesthesia type on surgical outcomes.

This study has several limitations. Firstly, the retrospective design of this study may introduce selection bias and limits the ability to establish causal relationships. In addition, patients requiring open reduction were excluded from the analysis. Therefore, more technically demanding fractures may have been underrepresented, which may have introduced additional selection bias. Additionally, although all radiographic measurements were performed by two trained orthopedic surgeons, inter-observer variability cannot be completely excluded, particularly in assessing reduction quality and implant positioning. Another limitation is that postoperative coronal alignment was categorized according to absolute postoperative CCDA values rather than changes relative to the contralateral hip or the patient’s native femoral alignment. Because the native femoral neck-shaft angle may vary between individuals, contralateral CCDA measurements could provide a more individualized assessment of postoperative varus or valgus alignment. However, standardized contralateral hip radiographs were not available for all patients in this retrospective cohort. Another limitation is that obesity was defined using BMI, which may not directly reflect local peritrochanteric soft tissue thickness. Local soft tissue thickness around the greater trochanter may be more directly related to fluoroscopic visualization, reduction difficulty, and implant positioning. However, CT-based measurements of local soft tissue thickness or skin-to-bone distance around the greater trochanter were not available for all patients in this retrospective study. Furthermore, as the study was conducted at a single institution, the findings may not be fully generalizable to other centers with different surgical teams, implants, or postoperative protocols. Moreover, patient-reported outcomes such as the Harris Hip Score may be influenced by subjective factors and adherence to rehabilitation, which were not strictly controlled. Finally, the relatively small sample size in certain subgroups, particularly obese patients with AO/OTA 31A3 fracture patterns, may limit the statistical power for subgroup-specific analyses. Therefore, separate subgroup or sensitivity analyses excluding AO/OTA 31A3 fractures were not performed, and fracture type was instead adjusted for in the multivariable regression model. Future prospective, multicenter studies with larger cohorts are warranted to validate these findings and further investigate the impact of obesity on surgical outcomes following PFN fixation of intertrochanteric femoral fractures.

## Conclusions

This study highlights the impact of obesity on surgical technique and outcomes in intertrochanteric femoral fractures treated with proximal femoral nailing in the lateral decubitus position. Our findings indicate that reduction is more challenging in obese patients compared with non-obese patients, although acceptable reductions can generally still be achieved. Obesity was associated with poorer reduction quality, longer operative time, and a higher risk of complications such as V-effect–related varus malposition. Surgeons should be aware of these technical challenges when treating obese patients and consider them during preoperative planning and intraoperative reduction maneuvers.

## Data Availability

All data supporting the findings of this study are available within the paper.

## Abbreviations

IFFs: Intertrochanteric femoral fractures
PFN: Proximal femoral nail
AO/OTA: AO Foundation/Orthopaedic Trauma Association
TAD: Tip–apex distance
CCDA: Caput-collum-diaphyseal angle
ICU: Intensive care unit
BMI: Body mass index
CCI: Charlson Comorbidity Index
